# Successful short interval staged surgery in synchronous type A aortic dissection and giant abdominal aortic aneurysm patient

**DOI:** 10.1016/j.heliyon.2022.e10964

**Published:** 2022-10-06

**Authors:** Zhipeng Hu, Zhiwei Wang, Xinping Min, Bowen Li, Min Zhang, Feifeng Dai, Xin Cai

**Affiliations:** Department of Cardiovascular Surgery, Renmin Hospital of Wuhan University, 238 Jiefang Road, Wuhan City, 430060, China

**Keywords:** Aortic dissection, Abdominal aortic aneurysm, Staged surgery, Medical decision making, Case report

## Abstract

The surgical therapy of synchronous type A aortic dissection and abdominal aortic aneurysm is complex and rarely reported, especially, when the abdominal aortic aneurysm is unsuitable for intervention. Recently, we have successfully performed sequential two staged surgeries on a 46-year-old woman. The first stage surgeries consisted of the Bentall procedure, total aortic arch replacement, and frozen elephant trunk implantation. The second stage surgeries included replacement from the descending aorta to the sub-renal abdominal aorta, reconstruction of the blood flow to the spinal, the celiac trunk artery, the left and right renal artery, the superior and inferior mesenteric artery, and the iliac artery of both sides. The interval between two surgeries was very short (42 days). The patient was recovered, and all functions of her body were reserved. According to our experience, it is feasible to prevent aortic rupture by short interval staged surgery in selected patients with synchronous type A aortic dissection and un-interventionable abdominal aortic aneurysm.

## 1 Introduction

Both type A aortic dissection (TAAD) and giant abdominal aortic aneurysm (AAA) are life-threatening diseases which need emergency treatment [1, 2]. When patient suffers from synchronous TAAD and giant TAA, we have to perform staged surgery because one stage surgery is too traumatic. However, the risk of aortic rupture exists throughout the therapy.

In this article, we report the detailed treatment process of a cured case. The patient has provided her written informed consent for the publication of this manuscript. The images and data have been de-identified.

## 2 Patient information

**Figure 1:**
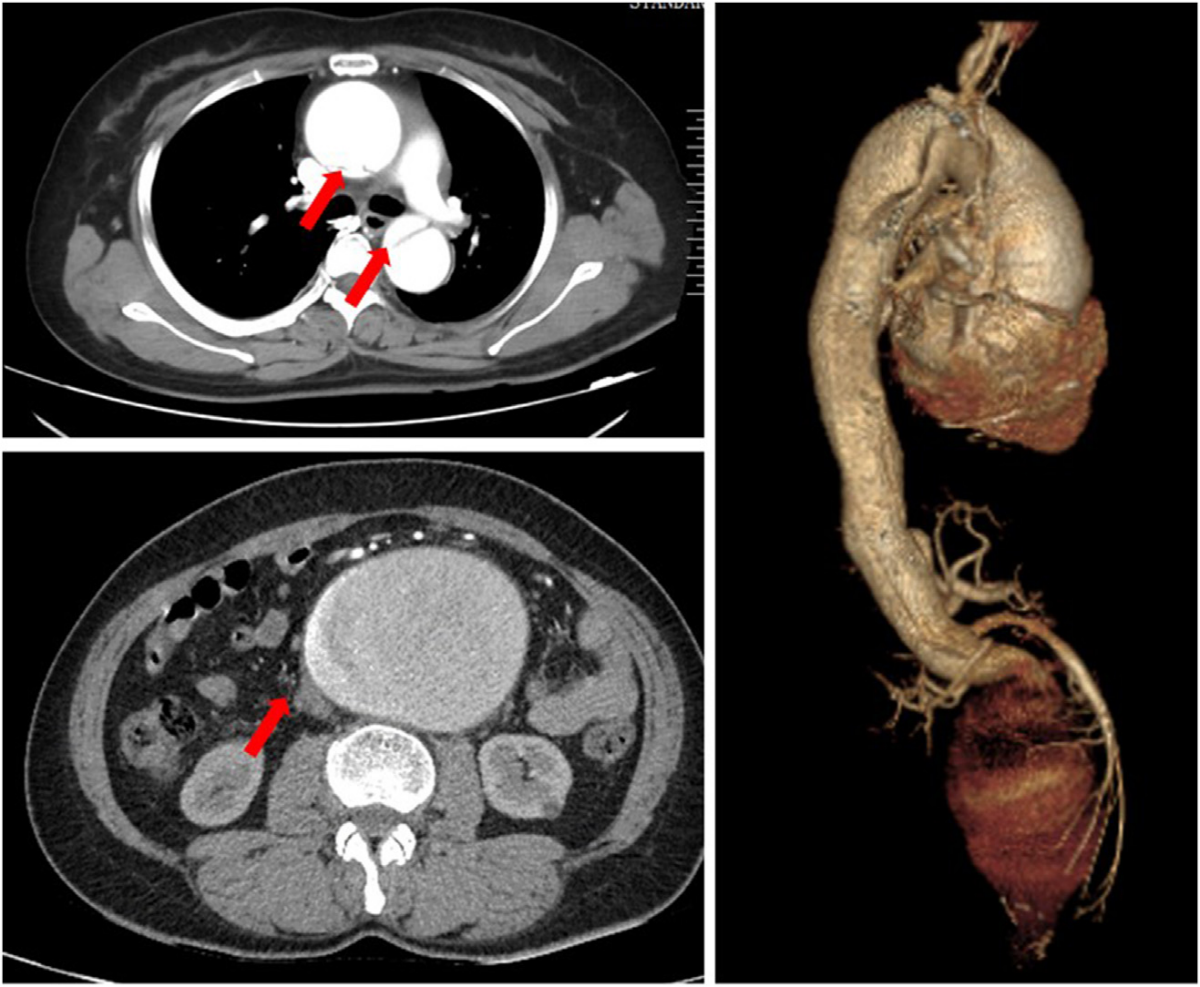
Pre-operative imaging of the patient.

A 46-year-old woman felt severe back pain suddenly. Computed tomography angiography (CTA) showed that she had suffered TAAD and giant AAA. Her aortic root was dilated to 41 mm. The maximum diameter of her AAA is 93 mm. Her aorta was dissected from the middle of her ascending aorta to her left iliac artery. Her celiac trunk artery and left renal artery were in the false lumen, while the superior mesenteric artery, inferior mesenteric artery, and right renal artery were in the true lumen of her dissected aorta (Figure 1). Intimal tears were found at the distal ascending aorta, the arch, the abdominal aorta right above the celiac trunk artery, and the body of the abdominal aortic aneurysm, and the primary entry was located at the proximal ascending aorta.

Echocardiography indicated a moderate to severe aortic insufficiency. She neither had clinical manifestations of Marfan syndrome nor a family history of hereditary aortic disease. Her blood pressure was normal then, and she did not have a hypertension history. She had not undergone any operation before.

On physical examination, there was a diastolic murmur in the second left intercostal space.

### 2.1 Treatment

#### 2.1.1 Surgical treatment of the thoracic aortic dissection (TAD)

Before median sternotomy, the right femoral artery and the right axillary artery were exposed for cannulation. The branches of the arch were isolated before opening the pericardium. After the cardiopulmonary bypass was running up, the body temperature was lowered to 28 °C. Then the aorta was clamped, and the ascending aorta was removed, followed by a Bentall procedure (23mm SJM Masters Series prosthesis, St. Jude Medical, Minnesota, USA). Under circulatory arrest and 15 l/min.Kg selective brain perfusion at 25 °C nasal temperature (the lowest nasal temperature was 25°C during the operation), a CRONUS stent (Shanghai Microport Orthopedics, shanghai, China) was placed into the true lumen of the descending aorta. The aortic arch was replaced with a branched prosthesis. Then, the ascending aorta prosthesis, the arch prosthesis, and the stent were connected.

#### 2.1.2 Strategy for bridging the first and the second surgery

We used a low tidal volume, low PEEP (Positive End Expiratory Pressure) strategy to maintain lower peak airway pressure. After detaching from the mechanic respirator, an ultrasonic expectorator was used to assist in clearing airway secretions clearing. Left lung atelectasis was diagnosed post-operatively, and bronchoscope therapy was done under intravenous anesthesia. The systolic blood pressure was strictly limited to 110 mmHg.

#### 2.1.3 Surgical treatment of the AAA

Forty-two days after the first surgery, a total thoracoabdominal aorta replacement was performed. After anesthesia, the left iliac artery was isolated. Then, an incision was made along the left sixth intercostal space and was extended to the left edge of the rectus abdominis muscle. The left arcus costarum was cut off at the sixth intercostal space. The abdominal aorta was isolated, and the integrity of the peritoneal cavity was maintained. One branch of the four-branched aorta prosthesis (Maquet, Rastatt, German) was connected to the left iliac artery. Then the descending aorta was clamped 2 cm above the distal end of the elephant trunk. The abdominal aorta prosthesis was connected to the distal end of the elephant trunk. Then, the aortic wall between the 8th to the 10th intercostal arteries was wrapped to a 0.5 cm diameter tube and was connected to one branch of the prosthesis. The clamper was moved down-ward to restore the blood flow to spinal arteries from the 8th to the 10th intercostal arteries. Then the distal end of the aorta prosthesis was sewed to the aorta wall including the celiac trunk artery, right renal artery, and the superior mesenteric artery. After the blood flow was restored to the above organs, the third branch of the aorta prosthesis was connected to the left renal artery. Finally, the last branch of the aorta prosthesis was connected to the right iliac artery and the inferior mesenteric artery in sequence. We did not use oxygenator, and normal body temperature was kept during the operation. Instead, we only used a roller pump of a CPB machine for blood recycling via a suction tube. After recycled to the blood stocker without oxygenator, the blood was then transfused into the body by another pump via a femoral artery cannula.

#### 2.1.4 Spinal protecting strategy

Prophylactic cerebrospinal fluid drainage was performed to prevent paraplegia. In detail, the drainage tube was placed before the second surgery. The cerebrospinal fluid pressure was restrained to lower than 10 cm H_2_O.

## 3 Results

**Figure 2:**
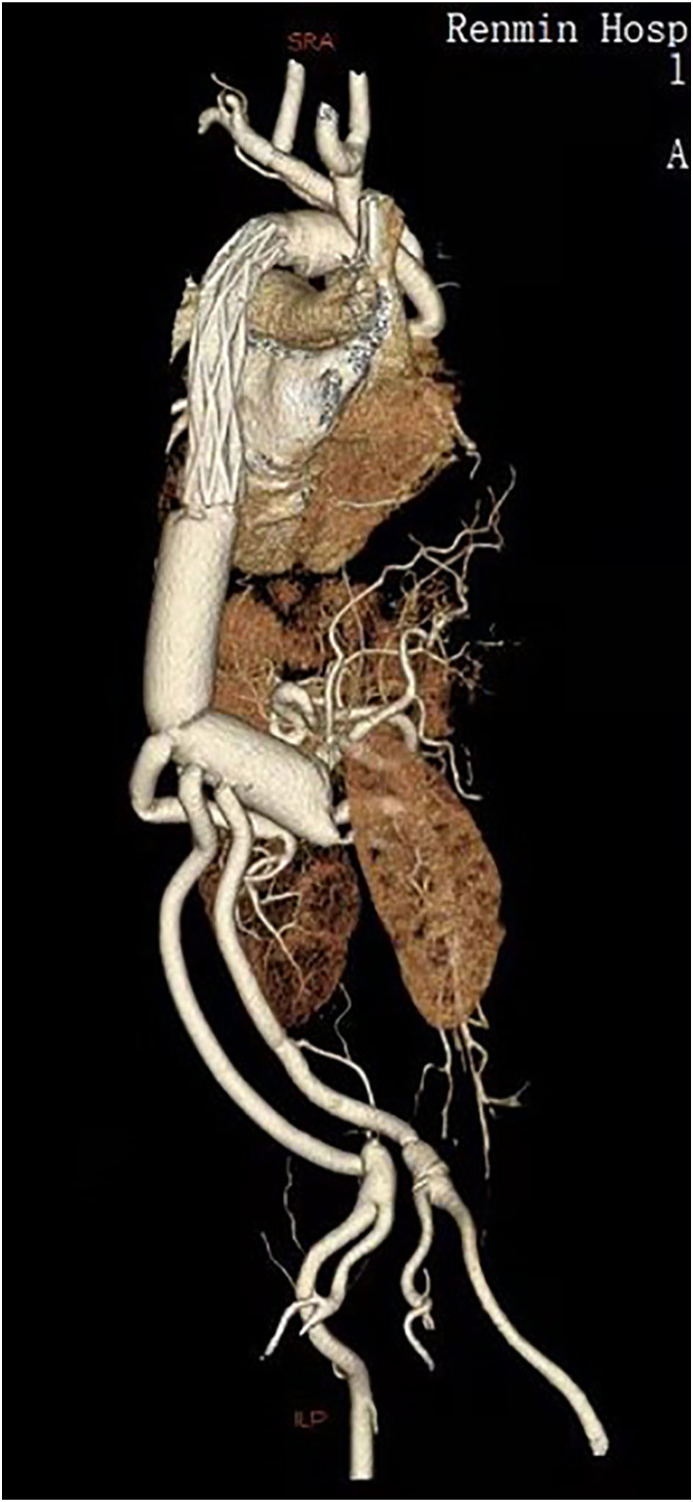
Post-operative CTA of the patient.

The total hospital stay is 61 days, and the ICU stay was 8 days. The red blood cell consumption was 20 u. The blood platelet consumption was 3 u. Mechanical ventilation time was 29 h for the first surgery and 37 h for the second surgery. Total cerebrospinal fluid drainage was 52 ml, and the drainage tube was pulled out 31 h after the second surgery. CTA was performed before discharge. Endo-leak was not found anywhere. All reconstructed branches of the aorta were patent except for the intercostal arteries (Figure 2).

The patient said that she had overcome her fear of death before discharge. She did not feel any discomfort from the aorta prosthesis.

## 4 Discussion

Currently, little information is available for synchronous TAAD and un-interventionable AAA in literature. Xu has reported a concomitant surgically cured case. However, that case is different from ours because the patient has been implanted with a stent in the descending aorta formerly, making him free from total arch replacement this time [3]. Even though, some experts debate the necessity of performing concomitant surgery in that case [4]. Because of the concern that this patient would die from the massive surgical trauma, we thought that she was not a candidate for one-stage surgery.

It is a dilemma to treat the TAD first or the AAA first. Whichever we choose to treat first, the other lesion is at a big risk of rupture. We finally chose to treat the TAD first for the following reasons: 1) TAD is more likely to rupture. 2) An elephant trunk will make the second surgery much more manageable [5].

The synchronous TAAD and AAA are more likely found in Marfan syndrome patients [6]. But most of these cases could be treated by staged surgery with several years’ interval. For example, Yuki Ikeno summarized 82 surgically treated Marfan syndrome cases, and reported that the average interval between the first and the second staged operations was 4.8 years [7]. Longer interval between operations means a longer time for the patients to recover and better tolerance to the second surgery. However, in this case, we did not have the opportunity to wait long after the first surgery.

A pity was that the reconstructed blood supply to the spinal artery via the intercostal arteries was not patent, most likely because of thrombogenesis, reminding us of the importance of postoperative anticoagulation therapy. Luckily, this case has not suffered paraplegia. This may partially benefit from our prophylactic cerebrospinal fluid drainage [8].

## Declarations

### Author contribution statement

All authors listed have significantly contributed to the investigation, development and writing of this article.

### Funding statement

This research did not receive any specific grant from funding agencies in the public, commercial, or not-for-profit sectors.

### Data availability statement

Data will be made available on request.

### Declaration of interests statement

The authors declare no conflict of interest.

### Additional information

No additional information is available for this paper.
